# The Human Periallocortex: Layer Pattern in Presubiculum, Parasubiculum and Entorhinal Cortex. A Review

**DOI:** 10.3389/fnana.2017.00084

**Published:** 2017-10-04

**Authors:** Ricardo Insausti, Mónica Muñoz-López, Ana M. Insausti, Emilio Artacho-Pérula

**Affiliations:** ^1^Human Neuroanatomy Laboratory, School of Medicine, University of Castilla-La Mancha, Albacete, Spain; ^2^Department of Health Sciences, Physical Therapy School, Public University of Navarra, Tudela, Spain

**Keywords:** human, entorhinal cortex, presubiculum, parasubiculum, layer pattern

## Abstract

The cortical mantle is not homogeneous, so that three types of cortex can be distinguished: allocortex, periallocortex and isocortex. The main distinction among those three types is based on morphological differences, in particular the number of layers, overall organization, appearance, etc., as well as its connectivity. Additionally, in the phylogenetic scale, this classification is conserved among different mammals. The most primitive and simple cortex is the allocortex, which is characterized by the presence of three layers, with one cellular main layer; it is continued by the periallocortex, which presents six layers, although with enough differences in the layer pattern to separate three different fields: presubiculum (PrS), parasubiculum (PaS), and entorhinal cortex (EC). The closest part to the allocortex (represented by the subiculum) is the PrS, which shows outer (layers I–III) and inner (V–VI) principal layers (*lamina principalis externa* and* lamina principalis interna*), both separated by a cell poor band, parallel to the pial surface (layer IV or *lamina dissecans*). This layer organization is present throughout the anterior-posterior axis. The PaS continues the PrS, but its rostrocaudal extent is shorter than the PrS. The organization of the PaS shows the layer pattern more clearly than in the PrS. Up to six layers are recognizable in the PaS, with layer IV as *lamina dissecans* between superficial (layers I–III) and deep (V–VI) layers, as in the PrS. The EC presents even more clearly the layer pattern along both mediolateral and rostrocaudal extent. The layer pattern is a thick layer I, layer II in islands, layer III medium pyramids, layer IV as *lamina dissecans* (not present throughout the EC extent), layer V with dark and big pyramids and a multiform layer VI. The EC borders laterally the proisocortex (incomplete type of isocortex). Variations in the appearance of its layers justify the distinction of subfields in the EC, in particular in human and nonhuman primates. EC layers are not similar to those in the neocortex. The transition between the periallocortical EC and isocortex is not sharp, so that the proisocortex forms an intervening cortex, which fills the gap between the periallocortex and the isocortex.

## Introduction and Historical Account

The great anatomists of the early 20th century (Vogt, 1903, cited in Triarhou, 2009) recognized that the human cerebral cortex was not homogeneous. Vogt (1903) named the six-layered cortex, isocortex (homogeneous cortex), which made much of the cortex in the brain. In contrast, “allocortex” (inhomogeneous, other, or strange cortex) lacked multiple neuron lamination. Ariëns-Kappers (1909) defined archicortex and paleocortex, which are “roughly identical” to the allocortex. The archicortex included the hippocampus and related structures, while paleocortex included what was called “*rhinencephalon*” (meaning olfactory brain). In this term both olfactory and hippocampal structures were considered together. Interestingly, while in other mammals the allocortical structures make up a great proportion of the cortical mantle, in humans that proportion is considerably reduced.

The concept of periallocortex (Pall) can be defined in classical neuroanatomy literature, dating back to the late nineteen and early twentieth centuries. Brodmann (1909) gave the first account of all Pall regions in man: Presubiculum (PrS), Parasubiculum (PaS) and entorhinal cortex (EC), and identified the main features of all of them.

The anatomical terms of “isocortex” and “allocortex” were introduced by Oskar Vogt in 1910 (cited in Stephan and Andy, 1970) and the purpose was to differentiate between the more common six-layered type of cortex vs. the uncommon, restricted to the rhinencephalon in broad sense, which one single neuron layer organization. For this reason, it was considered a more primitive-type of cortex. Filimonoff (1947) introduced the term periallocortex because it surrounded the allocortex, which was interposed between the isocortex and the allocortex. The allocortex itself was also divided into paleocortex, which corresponds to secondary olfactory centers, and archicortex, which is the hippocampus. Both, paleocortex and archicortex, present a peripheral region named peripaleocortex (i.e., periamygdaloid cortex and anterior insular related structures) and periallocortex which comprises the PrS, PaS and EC (Table 1). From the classification of the cerebral cortex in allocortex and isocortex derived the commonly used concepts of neocortex (new cortex, opposite to archicortex or old cortex). Another type of cortex interposes in between the periallocortex and the isocortex, which is known as proisocortex. The term proisocortex defines a type of cortex that does not fulfill all the layering features of isocortex, although is close (Bailey and von Bonin, 1951).

**Table 1:** Classification of the types of cortex.

3 layers	Oflactory Dentate gyrus, Hippocampus & Subiculum	Allocortex (archicortex)
6 layers (with LD)	Presubiculum & Parasubiculum Entorhinal cortex	Periallocortex
6 layers (without LD)	Proisocortex, i.e. perirhinal cortext
6 layers (with granular layer)	Isocortex (neocortex), i.e. association cortex

*The number of layers is indicated in each type: 3 layers for allocortex; 6 layers for periallocortex (including lamina dissecans); 6 layers for proisocortex (without lamina dissecans); 6 layers for isocortex (internal granular layer without lamina dissecans)*.

The layer organization of the three separated fields belonging to the periallocortex will be addressed in this review for its structural relevance and functional meaning in spatial navigation and memory. The connectional relationship of the different layers with other brain centers will be briefly addressed in this review (for more details see Insausti et al., 2017).

A common feature of all components of the periallocortex is the presence of a cell free zone, parallel to the pial surface, which receives the name of *lamina dissecans*. The presence of this cell free layer “splits” the thickness of the cortex into approximately equal halves. For this reason, Rose (1927) denominated these fields as *“schizocortex”*. This proposal is substantiated by the fact that the PrS, PaS and EC are present in all mammals, and all are principal components of the hippocampal formation.

## Common Features of the Periallocortex

**Figure 1:**
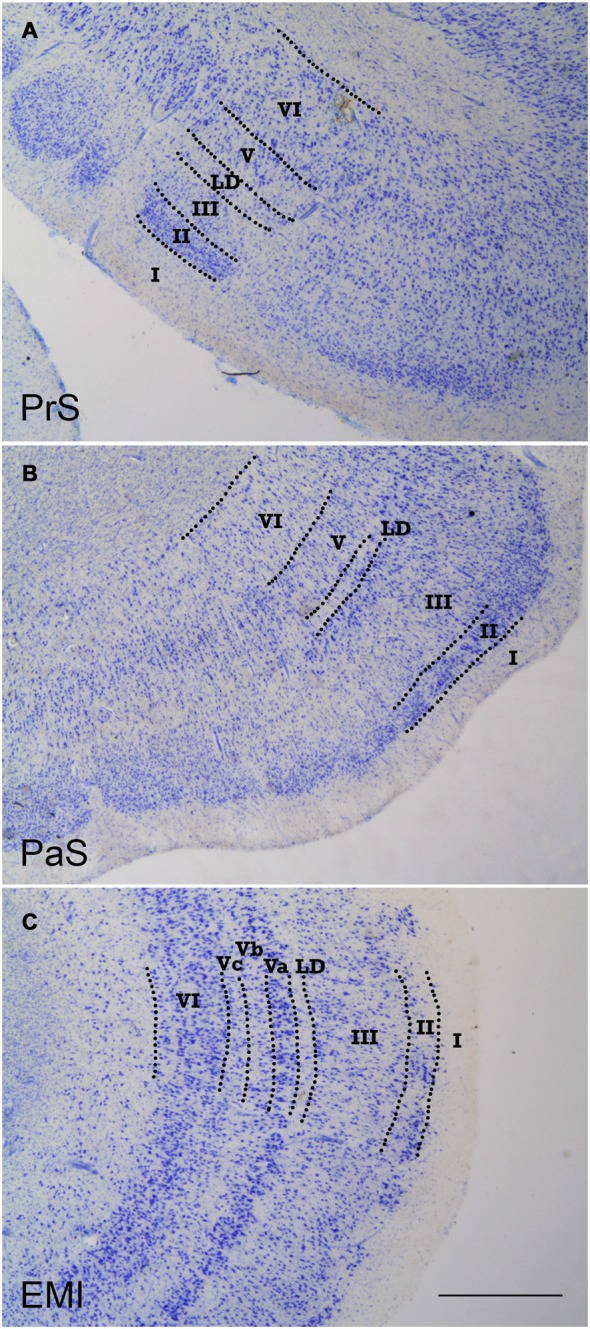
Low-power photomicrographs of coronal sections that show the three components of the periallocortex. **(A)** shows a coronal section of the presubiculum (PrS). The layer pattern is indicated in roman numerals. **(B)** is the parasubiculum (PaS), equally depicting the morphological features and layer pattern. **(C)** shows a representative section of subfield E_MI_ of the entorhinal cortex (EC). The layer pattern is equally demarcated by the broken lines. The similitudes and differences of the progressive differentiation of the layer pattern can be appreciated, from the more rudimentary in the PrS, to more developed in the PaS, to the maximal differentiation of layers in the EC. Note the constant presence of *lamina dissecans* in all three components of the periallocortex. Scale bar 500 μm.

Figure 1 shows a general representation of the typical appearance of the PrS (A), PaS (B) and EC (C), the three periallocortical fields. The main and distinctive characteristic of the periallocortex is the presence of higher number of layers than the allocortex. The periallocortex boundaries are with the allocortex represented by the subiculum, and with the proisocortex, present in the cortex lining the collateral sulcus. Here, the lateral boundary of the periallocortex forms an interface with either the perirhinal cortex^1^ or the posterior parahippocampal cortex (areas TH and TF of von Economo and Koskinas, 1925). A similar organization is present in a number of species (Ramón y Cajal, 1893; Brodmann, 1909; Lorente de Nó, 1933, 1934; Bakst and Amaral, 1984; Amaral et al., 1987; Insausti et al., 1995).

Probably the most common feature of the periallocortical cortex is the presence of a cell free band, halfway in the thickness of the cortex, which is named *lamina dissecans* (from latin, *dissecare*, to dissect), which dissects (splits) the cortex into two main layers, an external between the piamater, and the *lamina dissecans*, and an internal one, between *lamina dissecans* and the white matter. Although *lamina dissecans* is far from homogeneous, it is nonetheless present in all three fields of the periallocortex. The laminar structure and nomenclature of the periallocortex has been subject of debate, in particular the EC, which is the most laminated of all the Pall structures (for more details see Amaral et al., 1987).

## Fields of the Periallocortex

The distinction among the three fields of the periallocortex is based on morphological differences (number of layers, overall appearance), and connectional-functional significance. As mentioned above, Brodmann (1909) gave the first account of all the periallocortical regions in man: PrS, PaS and EC.

While the common feature of the allocortex is the presence of one neuron layer (i.e., dentate gyrus, hippocampal fields), the periallocortex shows several more layers. Of note, those layers are not similar to the layers in the neocortex, although we will use a similar sequence of roman numerals, meaning merely the order in which they appear, counting them from the pial surface to the underlying white matter.

The subtle transition of layers is most noticeable at the junction of the EC (periallocortex) and the proisocortex. The proisocortex marks the transition between periallocortex and the isocortex. The proisocortex shows six layers, as in the isocortex, but retain some of the periallocortical features such as prominent layers II and V, the lack or a thin layer IV, and an overall lesser columnarity than the isocortex. The proisocortex is largely coincident with the paralimbic cortex.

## Periallocortical Fields

### Presubiculum

The PrS has been clearly identified since the second half of the nineteenth century. However, its detailed structure in human and nonhuman primates has been rather fragmentary (Insausti and Amaral, 2012; Ding, 2013). Brodmann (1909) assigned, the number 27 to the PrS among the numbers he labeled the cortical areas. In his depiction of the medial surface of the human brain, this area runs in parallel to the hippocampus as far as the splenium of the corpus callosum, where it borders the retrosplenial cortex. He also noted the presence of the PrS in a number of nonhuman primates. Unfortunately, the description of the field and layers is almost nonexistent. von Economo and Koskinas (1925) provided a much more detailed account of the structure of the PrS^2^. According to their classic report, the PrS is characterized as a granular type of cortex (koniocortex). The layers that can be recognized in the human PrS, as well as in other mammals are:

1. Layer I or molecular layer. Thick and containing a great amount of fibers, whose origin is, in part, the EC through the perforant path.
2. Layers II and III, made up of rounded cells, which Ding (2013) calls pyramidal neurons, is subdivided into layers II and layer III. Both layers fuse together, with no clear boundary between both of them. Both layers II and III, are referred to by Braak (1980) as *lamina cellularis superficialis*, which is the nomenclature followed in this report.
3. Layer III, fused to the deep part of layer II. Layer III neurons are larger than its layer II counterpart.
4. Layer IV or *lamina dissecans*, which is one of the most characteristic features of the PrS, it separates the *lamina cellularis superficialis* from the *lamina cellularis profunda*, and divides the PrS into external and internal layers, approximately equal in thickness (0.61 mm outer, vs. 0.70 mm inner, excluding layer I, von Economo, 2009).
5. Layer V, is better identified at lateral portions of the PrS.
6. Layer VI, which has indistinct borders with layer V continues it as far as the limit with the white matter of the angular bundle.

A further layer (layer 7) has been identified in the monkey (Ding and Rockland, 2001), although it could be also a local extension of horizontal neurons of the internal part of the pyramidal cell layer of the subiculum.

There are almost no specific studies on the neurochemical phenotype of the human PrS; notwithstanding, partial information can be collected from different studies. In this sense, the immunoreactivuty for the calcium binding protein parvalbumin stands as the most remarkable, since the densely aggregated small neurons in the outer layers of the PrS are densely labeled (i.e., Figure 5 in Thangavel et al., 2008). The immunoreactivity of the PrS contrasts with the much lower immunoreactivity in the subiculum medially, and the PaS laterally.

**Figure 2:**
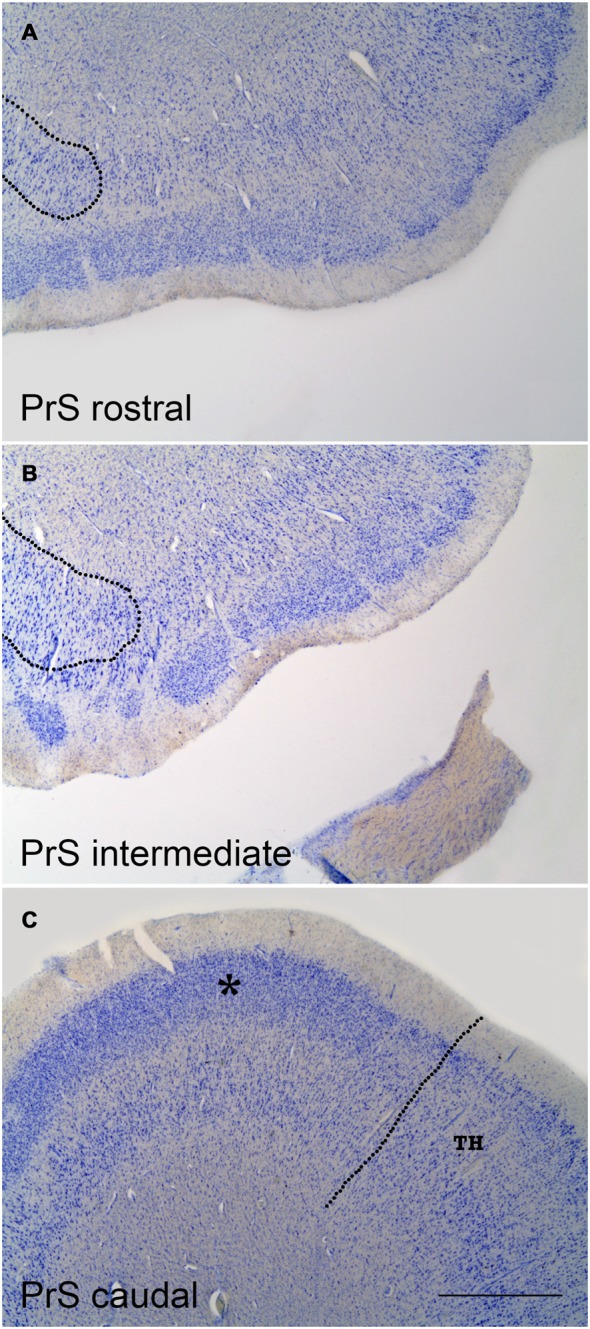
Coronal sections at three levels of the PrS as shown in low-power photomicrographs. **(A)** is at a very rostral level of the PrS; the rounded border with the distal subiculum is indicated by the broken line to the left. **(B)** is at midlevel of the PrS. Again, the border with the subiculum is indicated at the left; the overlap of the clumps of the PrS with the subiculum is evident. **(C)** is a coronal section through the caudal level of the PrS. The continuous band of layer II–III neurons (asterisk) is noticeable, as well as the border with area TH (von Economo and Koskinas, 1925) of the posterior parahippocampal cortex, which is indicated by the broken line. The layer pattern of PrS is not indicated for clarity, but it is similar to that indicated in Figure 1. Scale bar is 500 μm.

Figure 2 shows representative levels of the PrS at three different levels along the rostrocaudal axis. One of the most prominent and conspicuous features of the PrS layer II is the presence of clumps of small, rounded neurons, or aggregates of cells, amidst the white matter of layer I. Layer I is thicker in between clumps, in particular at the medial portion (closest to the subiculum). At this location, the PrS forms two or three conspicuous groups^3^ of small, densely packed and rounded neurons (granular appearance). Although the granular neurons are in layer II, it cannot be ruled out that some layer III neurons might be present. Seen from the surface, the PrS offers a lattice-like pattern, the *substantia reticulata alba* (Arnold, 1851), which can also be seen in the EC. Interestingly, the nonhuman primate PrS does not form islands throughout its extent, therefore this feature is exclusively present in the human brain. It is interesting to note that this organization of cell aggregates in layer II is also present at the caudal portions of the PaS and the EC, at precisely the same levels at which grid cells and head directions neurons have been described (Glasgow and Chapman, 2007; Miller et al., 2015; Suthana et al., 2015).

#### Rostrocaudal Variation of the PrS

According to Braak (1978, 1980) the small cells of layer II are “endogenous” to the PrS, while the deep layers mimic the adjacent fields, either subiculum proximally or EC distally. The latter is further caudally replaced by the posterior parahippocampal cortex (proisocortex).

Layers II–III appearance varies along the rostrocaudal axis of the PrS. Anteriorly, it breaks up into densely packed clumps of granular cells, while progressively the number of clumps decreases at posterior levels, where the PrS takes a more continuous appearance.

Of note, the PrS islands of small cells lie on top of the subiculum, and therefore the deep layers are limited to a small layer adjacent to the white matter. This portion of the PrS has also been considered as part of the subiculum (Braak, 1980), although laminar differences between both areas are obvious. However, the subiculum extends medially under these clumps and forms a termination of pyramidal neurons which form a rounded distal end of the subiculum (Figures 2A,B).

On tangential sections of the PrS, the arrangement of these clumps forms a lattice-like structure, not much different to the EC. The functional significance of this particular arrangement of layer II neurons of the PrS is unknown, although the possibility of interaction between presubicular layers on top and subicular layers underneath is intriguing.

Although the layering pattern of the PrS may be obscure at some levels, the layer organization is maintained throughout the anterior-posterior axis. A more complete account of the longitudinal variation of the PrS has been reported (Figure 1 in Braak, 1978).

The caudal extreme of the PrS continues with the granular portion of the retrosplenial cortex (BA 29) at the level of the *istmus* of the parahippocampal gyrus, which is also in the near vicinity of the rostralmost extreme of the calcarine fissure (Frankó et al., 2014). Here, layers II–III of the PrS form a more homogeneous association as a unique clump, and takes a continuous, elongated shape. The boundary with the granular part of the retrosplenial cortex (BA 29) is rather indistinct. The PrS extends as far as the end of the hippocampus.

No functional data on the specific activity of the PrS exist, although nonhuman primate studies show that the PrS is the main source of commissural, contralateral afferents to the EC (Demeter et al., 1985; Amaral et al., 1987; reviewed in Insausti et al., 2017). As direct commissural connections of the dentate gyrus and other hippocampal fields are scarce in human as well as in nonhuman primates, this feature becomes a clear species difference in the structural organization of the commissural hippocampal system relative i.e., to rodents. Connectional studies have also revealed that the PrS is one of the main non-entorhinal hippocampal output systems; connections with temporal cortices (in particular perirhinal cortex), frontal and parietal cortices have also been demonstrated (Barbas and Blatt, 1995; Blatt and Rosene, 1998; Ding et al., 2000; Insausti and Muñoz, 2001).

#### Comparison with the Nonhuman Primate

The demonstration of acetylcholinesterase (AChE) in the nonhuman primate brain gives further support for a layering pattern of the PrS very similar to humans. Bakst and Amaral (1984) study describe in the *Macaca fascicularis* monkey an outer layer I, the molecular layer, followed by the external principal layer made up of a dense, continuous band of small neurons. The lamina dissecans stands clearly, deeper to which the lamina principalis interna lies. This layer is much less stained than the lamina principalis externa, and contains a population of polymorphic neurons, without any specific orientation. While the outer layers of the PrS are easily identified, the deep layers present blurred boundaries with the adjacent subiculum and PaS.

### Parasubiculum (Figure 3)

The PaS continues the PrS towards the midline; it occupies the ventral shoulder of the hippocampal fissure. The PaS parallels the PrS for most of its course, although its rostrocaudal extent is shorter than PrS. The PaS starts a little caudal to the beginning of the PrS, and borders laterally the medial part of the EC. At caudal levels to the EC, area TH of von Economo and Koskinas (1925) forms the lateral boundary.

**Figure 3:**
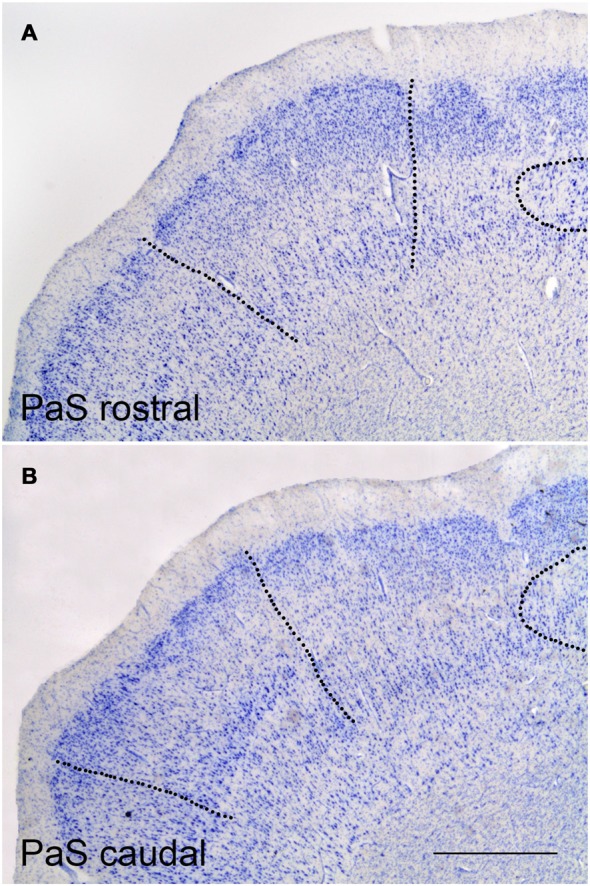
Representative low-power photomicrographs at two PaS levels. **(A)** shows a rostral coronal section where the borders with the PrS (right side) and caudal EC (left side) are indicated as broken lines Likewise, the rounded termination of the subiculum under the clumps of the PrS can be appreciated. **(B)** shows the PaS at a caudal level. The border of PaS with the PrS and the distal end of the subiculum (to the right side), and with area TH (to the left side), are indicated by broken lines. Scale bar is 500 μm.

While the overall laminar organization of the PaS keeps some similarities with the PrS, the laminar organization of the PaS is more complex than in the PrS. The number of layers present increases to up to five layers; a *lamina dissecans* extends between the superficial and deep layers, similar to the PrS. The layers than can be distinguished are:

1. Layer I or molecular layer. It is smooth, and presents no specific feature.
2. Layer II is made up of more widely spaced pyramids and larger than the granular neurons in layer II of the PrS.
3. Layer III is made up of rounded medium or small neurons, some pyramids and other neurons with variable shape, whose boundary with layer II is rather indistinct.
4. *Lamina dissecans* is ill defined and discontinuous at some points.
5. Layer V lies beneath *lamina dissecans* and intermingle with layer VI of the EC, caudal to the start of the hippocampal fissure, (see below), although the large, deeply stained large pyramids in layer V of the EC are missing in the PaS.
6. Layer VI has no clear boundary, neither with layer V nor with the white matter. Laterally, at the transition with the EC, layers V and VI of EC seem to be in continuation with the PaS, although the latter shows more variety in the morphology and orientation.

The PaS extends behind the EC caudally, although the boundary is far from being smooth. Instead, in series of coronal sections through the end of the EC clumps of small layer II neurons intermingle with the PaS. In nonhuman primates, however, the PaS forms a continuous band that surrounds caudally the extent of the EC (Amaral et al., 1987). In humans, the PaS does not form a continuous band at the transition between the caudal end of the EC and the field TH of von Economo and Koskinas (1925), but an intermingling of layer II islands of the caudal pole of the EC and layer II PaS neurons, a feature that brings complexity to this part of the PaS. Eventually, the PaS recedes and continues approximately as far as the beginning of the caudal part of the PrS.

The information related to the neurochemical phenotype of the human PaS specifically is almost non-existent, although scattered data can be gathered in the literature (Thangavel et al., 2008).

In the nonhuman primate, the laminar structure of the PaS is basically similar to the PrS (Bakst and Amaral, 1984). The outer part of the PaS contains a molecular layer and an outer layer, which can be subdivided into a more densely packed outer band that covers approximately 25% of its depth, while the deep portion contains rounded or pyramidal neurons, more evenly spaced and less dense than the outer 25% (Bakst and Amaral, 1984). The lamina dissecans is present, although it is much less conspicuous than in the EC or the PrS. The deep portion contains larger neurons, which show little staining density in Nissl stain. Acetylcholinesterase staining preparations reveal a high density of the reaction product in the outer cell band of the PaS.

### Entorhinal Cortex (Figure 4)

The human EC extends for a sizeable surface on the anterior part of the medial temporal lobe, in the anterior part of the macroscopically defined parahippocampal gyrus *(Gyrus parahippocampalis)*. In this location the EC borders rostrally the perirhinal cortex BA 35 (PRC, proisocortex) and amygdaloid complex. The PRC continues back laterally to the EC, in the medial bank of the collateral sulcus. In this location related to the collateral sulcus, PRC accompanies the whole rostrocaudal extent of the EC (Insausti et al., 1998; Ding and van Hoesen, 2015). Medially, the limits of the EC are clear, first, with the amygdaloid complex by the *sulcus semiannularis*, and, once the hippocampal fissure is present, the caudal boundary is with the PaS for about the caudal one-half of the EC (Insausti et al., 1995).

**Figure 4:**
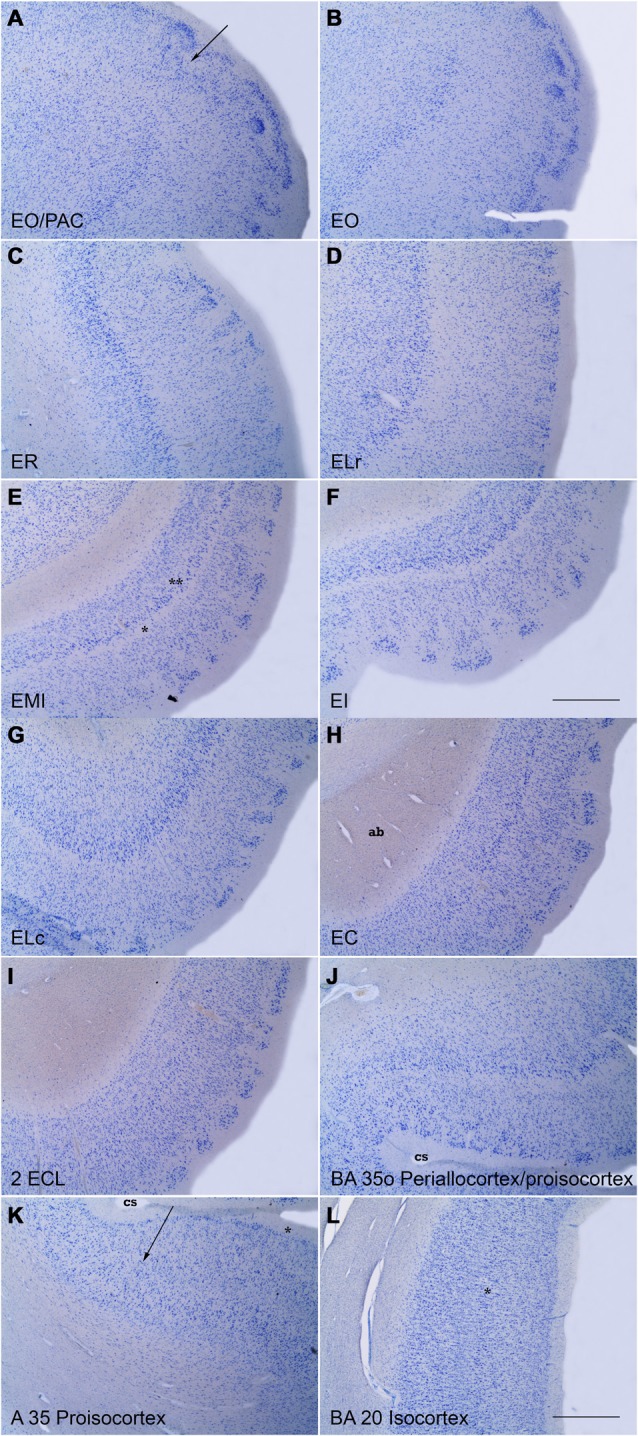
Series of low power photomicrographs of coronal sections throughout the different subfields of the EC and adjacent proisocortical and isocortical cortices. **(A)** is at the transition between the rostral part of the EC (subfield E_O_) and the amygdaloid complex (periamygdaloid cortex) The arrow indicates the border between the two. **(B)** is a representative section of subfield E_O_. Note the absence of *lamina dissecans* and the characteristic *lamina cellularis profunda*. **(C)** shows subfield ER. Note the organization of layer III neurons in clusters. The bottom of the panel shows the transition with subfield E_LR_. **(D)** is a representative section of subfield E_LR_. Note the wide space (III–V interval), characteristic of this subfield. **(E)** is subfield E_MI_. Note the sharpness of *lamina dissecans* (asterisk) and sublayer Vc (double asterisk). **(F)** is subfield E_I_. Note the clear layer pattern of layer II islands, and a clear *lamina dissecans*. **(G)** is subfield E_LC,_ which occupies the shoulder of the lateral bank of the collateral sulcus (cs). Note the likeness with subfield E_LR_. **(H)** is a representative level of subfield E_C_. Note the absence of *lamina cellularis* *profunda* as a neat border with the white matter of the angular bundle (ag). **(I)** shows subfield E_CL_. Note layer II islands surrounded by white matter, the columnarity of layers III to VI, and the neat border with the white matter. **(J)** shows the oblique transition between the lateral part of EC (subfield E_LC_) and the proisicortex of BA 35 (transentorhinal cortex of Braak and Braak, 1985). **(K)** shows respectively the transition between BA 35 and 36 (arrow) at the lateral bank of the collateral sulcus (asterisk). **(L)** is an example of isocortex with an evident inner granular layer IV (asterisk). No delimitation of the layers is indicated for clarity. Scale bar in all layers is 500 μm.

Along its extent the EC presents a clear six -layered pattern. The number and names of the layers have been changing along the years, but the common notion of six (or seven) layers predominates. (for more details see Amaral et al., 1987). Layering in the EC has been best observed with aldehyde fuchsin stain in thick sections (800 μm), as reported by Braak (1972, 1980).

Despite this common pattern, substantial differences exist along its mediolateral and rostrocaudal extents of the EC. Those differences have been taken into account, and historically, different terminology and number of subfields have been described (Sgonina, 1938; Macchi, 1951; Braak, 1980; De Lacalle et al., 1994; Insausti et al., 1995; Krimer et al., 1997). Based on the peculiarities in the mediolateral and rostrocaudal extent of the EC, we proposed up to eight subfields, which are architectonically very similar to the subfields proposed in the nonhuman primate (Amaral et al., 1987).

The specific features of the different EC subfields have been reported previously in detail (Insausti et al., 1995). However, we present here the general organization and laminar particularities of each subfield. It needs to be taken into account that although the layers of the EC have been named layers I to VI, they are not homologous to neocortical layers I to VI. For instance, layer IV in the cortex does not correspond to layer IV in the EC (inner granular layer vs. a cell-free band).

#### Olfactory Subfield (E_O_)

This subfield is located at the rostral-most portion of the EC. In nonhuman primates receives direct olfactory afferents from the olfactory bulb. The layer organization can be described as follows:

1. Layer I, or molecular layer is wide.
2. Layer II is thin, and broken up into two or three narrow islands
3. Layer III contains medium size, pale neurons homogeneously distributed
4. No *lamina dissecans* present
5. Layer V is indistinct and fuses with layer VI
6. Layer VI is wide and extends deep into the white matter. For this reason, it receives the name of *lamina cellularis profunda* (Braak, 1980)

#### Rostral Subfield (E_R_)

E_R_ subfield borders medially subfield E_O_. E_R_ makes up much of the anterior portion of the EC. Laterally it continues with subfield EL_R_. The pattern of layers in subfield E_R_ is:

1. Layer I, wide, but without specific features. Some little prominences called *verrucae hippocampi* or warts, make the surface uneven (Klingler and Gloor, 1960; Simic et al., 2005).
2. Layer II is discontinuous, and forms small, rounded islands of neurons.
3. Layer III is typically organized into clusters of small or medium pyramids, which are separated by cell-poor spaces. The inner part of the layer shows a more continuous appearance.
4. No *lamina dissecans* is present at rostral levels; at a more caudal level, a thin band of low cellularity separates layers III and V.
5. Layer V is distinguished by the presence of a continuous band of larger, deeply stained neurons.
6. Layer VI contains neurons of various sizes and shapes. There is no distinct border with the white matter, and the inner part of layer VI enters for some distance into the white matter. This feature has been named by Braak (1980) *lamina cellularis profunda*, which interestingly is not present at more caudal subfields of the EC.

#### Lateral Rostral Subfield (E_LR_)

This subfield occupies much of the lateral surface of the anterior one-half of the EC. E_LR_ borders medially E_R_, and laterally its boundary is marked by the transentorhinal cortex (Braak and Braak, 1985), which is a subdivision of BA35 or (PRC, proisocortex). The laminar features of subfield EL_R_ are:

1. Layer I, shows a smooth appearance (much fewer *verrucae hippocampi*).
2. Layer II is thick and broken into wide islands.
3. Layer III presents homogeneous appearance of medium sized pyramids.
4. Layer IV is wide and makes a clear separation between layers III and V. Myelin stain reveals a dense mesh of fibers that occupies the space. This feature is maintained all along the subfield and it is distinct to, and present at levels where the *lamina* *dissecans* has not appeared yet. This layer has been named “III–V interval” (Insausti et al., 1995).
5. Layer V is thick and prominent and invested with large pyramids, densely stained in Nissl preparations.
6. Layer VI contains neurons of various sizes and shapes and lacks a *lamina cellularis profunda*.

#### Intermediate Subfield (E_I_)

This subfield is situated midway in the EC. It is usually presented as the most typical level of the EC in which all layers of the EC are clearly shown (Braak, 1972, 1980). E_I_ borders medially subfield E_MI_, while laterally it limits with E_LC_. The layer features of this subfield are as follows:

1. Layer I, is wide and the surface presents a bumpy appearance due to the presence of the *verrucae hippocampi*, which can be appreciated to the naked eye (Simic et al., 2005; Insausti and Amaral, 2012).
2. Layer II is discontinuous by the presence of a neat layer II islands of dark stellate neurons.
3. Layer III is irregular at the limit with layer II, but more homogeneous at the inner part, and forms a neat line with layer IV.
4. Layer IV has the appearance of typical *lamina dissecans*, which shows an almost complete absence of neurons, forming a neat line with both layer III and layer V.
5. Layer V is made up of large pyramids which are organized into three sublayers. The outer part (sublayer Va) borders *lamina dissecans*, and it displays dense concentration of pyramids. Sublayer Vb lies underneath, and contains a lesser density of pyramids. Finally, the innermost is sublayer Vc, which contains a low density of neurons that forms a cell-poor band at the boundary with layer VI.
6. Layer VI is formed by homogeneous pyramids, which are densely packed. In contrast with more rostral levels, layer VI lacks *lamina cellularis profunda*, and shows a clear boundary with the white matter.

#### Medial Intermediate Subfield (E_MI_)

This subfield is coincident with the *Gyrus ambiens*, and is located at the dorsomedial part of the EC, immediately behind the olfactory subfield (E_O_). This subfield is very noticeable as it lies between the *sulcus semiannularis* dorsally, and the *sulcus intrarhinalis* (Insausti and Amaral, 2012) which ends at the rostral tip of the hippocampal fissure. The laminar organization of the subfield E_MI_ shows the representative layer organization of the periallocortex even more clearly than subfield E_I_. The medial part of E_MI_ shows all the layers with a compact appearance, while the lateral part resembles subfield E_I_. The *sulcus intrarhinalis* ends at the caudal part of the subfield E_MI_, at the point where the hippocampal fissure first appears. Then, the *Gyrus ambiens* (E_MI_ subfield) is replaced caudally by the *Gyrus uncinatus*, which is the transitional zone between the amygdaloid complex and the hippocampus. The layer organization of the subfield E_MI_ is:

1. Layer I is thinner relative to the adjacent subfield E_I_ and smooth (few or no *verrucae hippocampi*).
2. Layer II is thin and more continuous than the adjacent subfield E_I_. In this respect, it resembles more layer II of subfield E_O_.
3. Layer III is compact and homogeneous with medium pyramids evenly distributed.
4. Layer IV is *lamina dissecans*, and it presents a neat appearance.
5. Layer V is narrow. Sublayer Va is made up of big and dark pyramids. Sublayer Vb is indistinct. Sublayer Vc is very prominent and appears as a cell-free band that clearly separates sublayers Va and Vb from layer VI, and parallels *lamina dissecans*. This is a unique feature in all subfields of the EC, and an unmistakable feature of the subfield E_MI_.
6. Layer VI is narrow and compact. At the medial extreme of the layer, it fuses with the overlying hippocampo-amygdalar transitional area (HATA) area (Rosene and van Hoesen, 1987).

#### Lateral Caudal Subfield (E_Lc_)

This subfield continues caudally the subfield E_LR_. The posterior end of E_LC_ subfield takes place approximately at the level at which the hippocampal fissure is present. The layer organization of this subfield E_LC_ is:

1. Layer I, is wide with little indication of the presence of *verrucae hippocampi*.
2. Layer II has fewer, thick islands of stellate neurons.
3. Layer III presents medium-size pyramids, skewed towards the adjacent transentorhinal cortex.
4. Layer IV is made up of a thick mesh of fibers, which is narrower than layer III-V interval in subfield E_LR_.
5. Layer V is thick and shows loosely arranged pyramids.
6. Layer VI is indistinct and continues with layer VI of the transentorhinal cortex without any clear border.

#### Caudal Subfield (E_C_)

This subfield is in direct continuation with subfield E_I_. While the transition between E_I_ and E_C_ is gradual, the presence of the hippocampal fissure is an indication of the boundary between these adjacent subfields. Therefore, E_C_ subfield occupies the part of EC immediately caudal to the opening of the hippocampal fissure. E_C_ subfield spans from the PaS (or PrS very rostrally) to the transentorhinal area, at the medial bank of the *sulcus collateralis*. The main layer features of subfield EC are:

1. Layer I, is thick and presents numerous *verrucae hippocampi*.
2. Layer II is invested with clearly separated cell islands made up of big, stellate neurons, which show a rounded or polygonal appearance. The upper limit of layer II islands corresponds to the *verrucae hippocampi* present on the surface of the subfield. The inner part of layer II is isolated from the underlying layer III by a dense stratum of fibers.
3. Layer III is a homogeneous stratum of medium-to-big pyramidal neurons, which organize in a radial, columnar fashion.
4. Layer IV is *lamina dissecans*, although much less prominent than in subfields E_MI_ and E_I_.
5. Layer V is formed by big pyramids, homogeneously distributed. Sublayers can be recognized although sublayers Va and Vb tend to fuse; in contrast, sublayer Vc is increasingly wider and more evident; this is a feature that may confound with *lamina* *dissecans*, although the location of this sublayer is under the pyramidal neurons of layer Vc-b, and not above them, under layer III.
6. Layer VI is thick and presents a sharp boundary with the underlying white matter.

#### Caudal Limiting Subfield (E_CL_)

This subfield forms the caudal-most portion of EC. Subfield E_CL_ spans from the indistinct boundary with subfield EC as far as the transition with the PaS and its caudal continuation with the medialmost part of area TH (posterior parahippocampal cortex, von Economo and Koskinas, 1925). This subfield is as wide as subfield EC anteriorly, but progressively decreases in breadth. The medial border is coincident with the lower lip of the hippocampal fissure, while the lateral boundary is the posterior part of the transentorhinal cortex. The layer organization is:

1. Layer I, is thick and progressively presents fewer *verrucae hippocampi*. This surrounds completely layer Layer II islands.
2. Layer II is made up of neat cell islands with no significant difference with layer II islands of subfield E_C_.
3. Layer III presents a very columnar appearance, as neat and radial columns of medium pyramids. The outer part of the layer is adjacent to the mesh of fibers which surrounds layer II, thereby, in Nissl preparations, a cell free band interposes between layers II and III.
4. Layer IV, *lamina dissecans*, is absent so that layers III and V fuse together.
5. Layer V is made up of large pyramids, also radially oriented. The upper limit of the layer is fused with layer III, the only difference being the size and staining density of the pyramids, more pronounced in layer V, although they become progressively more similar. Sublayers Va and Vb cannot be distinguished, and make a single sublayer. However, sublayer Vc increases in width, and it is often mistaken with *lamina dissecans*.
6. Layer VI is also homogeneous, and presents a sharp border with the white matter of the angular bundle.

There are more data on the neurochemical phenotype or receptor layer distribution of different populations of the EC, although they are usually restricted to a sample or specific subfield as detailed above (Solodkin and van Hoesen, 1996, and Palomero-Gallagher and Zilles, 2017). From the structural point of view different techniques ranging from histochemical stains to receptor ligand demonstration demonstrate differences in the density of staining across different layers.

In this regard, it is important to note that histochemical staining for the demonstration of acetylcholinesterase reveals that the staining density is higher in the upper layers (Solodkin and van Hoesen, 1996). Likewise, the density of the enzyme cytochrome oxidase, which is related to energy demand of neurons, also shows stain in the upper layers II and III, while the layers V and VI present much lower staining density (Hevner and Wong-Riley, 1992; Solodkin and van Hoesen, 1996). Immunohistochemical demonstration of peptides have also been reported (i.e., somatostatin_28_, Friederich-Ecsy et al., 1988; Solodkin and van Hoesen, 1996), its distribution being denser in layer II stellate cells and pyramids of layers III and V. Likewise the distribution of neuropeptide Y (NPY) was mainly located in layers III and V. It is worth noting that the distribution of different staining methods yield an arrangement as “modules” pattern (Solodkin and van Hoesen, 1996).

An interesting approach, although seldom used, is the analysis of receptor distribution in the EC, of which there is a very recent report (Palomero-Gallagher and Zilles, 2017). In this report, the distribution of several receptors is reported in a very small portion of subfield γ15 of Sgonina (1938), which corresponds to a sample of subfield E_LR_ in more recent studies on the subfields of the EC (Insausti et al., 1995). Regardless of the nomenclature and laminar terminology (it is used the layer terminology of Braak, 1980), it is interesting to note that it is mostly the upper layers (layers II and III of the present report, layers Preβ, Preγ, Preγ_1_, Preγ_2_, and Preγ_3)_, the layers which show a higher density of several receptors (subunits of GABA receptor, AMPA, A1, mGLUR 2/3, A1 M1, and β1). The deep layers (V and VI of the present report, layers Priβ and Priγ) show high density in kainate receptors, while the muscarinic receptor M_2_ is present in the deep portion of layer III (Preγ_2_ and Preδ). Interestingly, NMDA receptors do not show any particular layer distribution, and other receptors (nic β4γ_2_, D_1,_ and 5-HT_2_), show little density.

### Functional Implication

At the present, it is difficult to ascribe any given function to periallocortex layers (PrS, PaS, EC) as such. However, there is experimental and clinical evidence of the involvement of the periallocortex in memory (Suthana et al., 2015) and spatial navigation (Glasgow and Chapman, 2007; Miller et al., 2015).

The concept of periallocortex is a type of cortex which presents more layers than in the allocortex. This fact could be used as a guide for the separation of the subiculum (allocortex) from the periallocortex (PrS, PaS and EC), and at the same time, to suggest dropping the term “subicular cortex”, which includes both the PrS and PaS as incorrect, as it combines allocortical (subiculum) and periallocortica (PrS, PaS) fields. The information available on the structure of the periallocortex in the human brain is very limited in terms of neurochemical phenotype or presence of different receptors. However, the increasing number of studies about functional activity in the hippocampal formation and the medial temporal lobe in general, precise a clarification and substantiation of the anatomical terminology used in the ascription of a name to any particular activated brain region in the hippocampal formation. Therefore, a renewed interest is arising on the location, boundaries and extension of periallocortical cortices in the human brain in different physiological and pathological situations, singularly human memory and, on the pathological side, Alzheimer’s disease.

## Author Contributions

RI designed the format and wrote the manuscript. MM-L and AMI extended and revised the original manuscript. EA-P chose and prepared the photomicrographs of the manuscript.

## Conflict of Interest Statement

The authors declare that the research was conducted in the absence of any commercial or financial relationships that could be construed as a potential conflict of interest.

## Footnotes

^1^The term perirhinal cortex is used in a generic way, to denote the band of cortex that mainly borders laterally the entorhinal cortex, and encompasses both area 35 (true perirhinal cortex) and area 36, or ectorhinal cortex (Brodmann, 1909).

^2^The translated edition of Triarhou (2009) is used in this review.

^3^We denominate clump the conspicuous aggregates of layer II neurons in the PrS, while we reserve the term “island” to the formation of layer II neurons in the EC. Other names have been used in the past such as “clouds” (Braak, 1980)
